# The protective association of mindfulness non-reactivity with depression among clinical nurses: a cross-sectional study

**DOI:** 10.3389/fpsyt.2026.1897938

**Published:** 2026-08-05

**Authors:** Zhi-Wen Gu, Xin Qin, Chang-Ping Yang, Zi-Sheng Lin, Zi-Long Ye, Li-Ping Chen

**Affiliations:** 1 Department of Psychiatry, Guangzhou First People's Hospital, South China University of Technology, Guangzhou, Guangdong, China; 2 The Second Affiliated Hospital, Guangzhou Medical University, Guangzhou, Guangdong, China

**Keywords:** clinical nurses, depression, mindfulness, non-reactivity, sleep quality

## Abstract

**Background:**

Clinical nurses are essential to healthcare, but they experience high rates of depression, anxiety, and sleep disturbances. This study examined the relationships among occupational stressors, sleep problems, mindfulness, and psychological well-being in hospital nurses.

**Methods:**

In a cross-sectional study of 416 clinical nurses from a tertiary general hospital in China, we assessed mindfulness, depression, anxiety, sleep quality, insomnia, and work stress. Multivariate logistic and linear regression models explored independent factors associated with depression, focusing on mindfulness dimensions.

**Results:**

Depression was highly prevalent (56.0%), as were clinical insomnia (52.4%) and poor sleep quality (54.6%). Multivariate analysis identified poor sleep quality (OR = 4.19, 95% CI: 2.47–7.12), insomnia (OR = 2.15, 95% CI: 1.22–3.75), and high work stress (OR = 2.12, 95% CI: 1.25–3.60) were significantly associated with an increased risk of depression. Notably, of the five mindfulness facets, only non-reactivity associated with a reduced risk of depression (OR = 0.88, 95% CI: 0.79–0.98). This association was consistent, showing a significant linear association in multivariate linear regression (B=-0.509, *p<*0.001).

**Conclusion:**

This study highlights the severe mental health burden among clinical nurses and demonstrates mindfulness non-reactivity is significantly associated with a lower risk of depression. These findings provide a preliminary empirical basis for future intervention studies targeting mindfulness facets.

## 1 Introduction

Clinical nurses are critical to healthcare systems worldwide. However, this workforce faces a mental health crisis (1, 2). In China, a meta-analysis of 45249 nurses reported a depression detection rate of 43.8% (3), while a global meta-analysis found that depression and anxiety affected approximately 32% of nurses during the COVID-19 pandemic (4). Poor psychological well-being not only inflicts personal suffering but also compromises patient safety through increased medical errors (5) and contributes to high burnout and turnover rates, thereby threatening the stability of entire healthcare systems (6).

Consequently, mindfulness has gained attention as a promising psychological resource for enhancing resilience against occupational stress. Meta-analyses have confirmed that mindfulness-based interventions can reduce burnout and improve mental health outcomes among nurses (7–9). However, an important question remains. Mindfulness is a multifaceted construct comprising distinct dimensions such as observing, describing, and non-reactivity (10). It is unclear which specific dimensions most effectively protect nurses from adverse outcomes such as depression, limiting the development of targeted interventions.

This study used the Five Facet Mindfulness Questionnaire (FFMQ) (10) to determine which specific mindfulness dimension is independently associated with a reduced risk of depressive symptoms in clinical nurses, after rigorously controlling for key confounders including work stress, anxiety, and the pervasive issue of sleep disturbances. By employing multivariate regression models, we aimed to isolate the unique contribution of each facet, with a particular focus on testing the role of ‘non-reactivity to inner experience’.

Our study shifts the focus from whether mindfulness is associated with better outcomes to which specific facet correlated with reduced depressive symptoms, by identifying the core dimension of mindfulness linked to depressive symptomatology. Identifying non-reactivity as the key mindfulness dimension associated with lower levels of depressive symptoms provides a robust empirical basis for developing targeted, streamlined, and potent mindfulness training programs tailored specifically for nursing populations. Such facet-specific intervention design holds potential to protect nurses’ mental well-being, alleviate job-related psychological distress, which may contribute to reduced turnover intention (11) and sustained delivery of high-quality, safe patient care.

## 2 Methods

### 2.1 Study design and participants

This cross-sectional study was conducted from January to June 2025 in Guangzhou First People’s Hospital. Using convenience sampling, we invited 450 registered nurses from 12 clinical departments (internal medicine, surgery, pediatrics, ICU, emergency, obstetrics, etc.) to participate. Inclusion criteria: (1) registered nurses with ≥1 year of clinical work experience; (2) voluntary participation and ability to complete the questionnaire independently. Exclusion criteria: (1) nurses on long-term leave or internship; (2) those with serious mental or physical illness; or (3) incomplete questionnaires. After excluding invalid or incomplete responses, 416 valid questionnaires were included (effective rate: 92.4%). The final analytical dataset contained no missing values, and no imputation procedures were required.

*A priori* sample size calculation was performed via G*Power 3.1 for multiple linear regression. We planned a model containing 1 primary predictor and 14 covariates (15 predictors in total). Assuming a medium effect size f2=0.15, α = 0.05, and a target statistical power of 0.95, at least 199 valid participants were required. A total of 416 participants were finally recruited, providing sufficient statistical power to detect the hypothesized association.

This study was approved by the Ethics Committee of Guangzhou First People’s Hospital (Approval Number: k-2020-026). All participants provided electronic informed consent before enrollment. All data were anonymized and securely stored.

### 2.2 Instruments

#### 2.2.1 General demographic and occupational questionnaire

We used a self-developed questionnaire to collect sociodemographic data (including age, gender, marital status, education, professional title, years of service, and department type) and occupational factors (including shift work, night shift frequency, work interest, and perceived work stress). It also included lifestyle and health-related variables such as chronic disease, medication use, caffeine/alcohol intake, exercise habits, and sleep-wake schedule.

#### 2.2.2 Mindfulness

We measured mindfulness using the Five Facet Mindfulness Questionnaire (FFMQ, Chinese version), which includes 39 items assessing five dimensions: Observing, Describing, Acting with Awareness, Non-judging, and Non-reactivity. Each item is rated on a 5-point Likert scale (1 = never or very rarely true, 5 = very often or always true). Higher total and subscale scores indicate greater mindfulness. The Chinese version of the FFMQ has been widely used in Chinese populations and has demonstrated satisfactory psychometric properties in previous studies (10). In the present sample, the Cronbach’s α coefficients were 0.760 for Observing, 0.810 for Describing, 0.704 for Acting with Awareness, 0.684 for Non-judging, and 0.612 for Non-reactivity.

#### 2.2.3 Depression

We assessed depression using the Beck Depression Inventory (BDI), which includes 21 items scored from 0–3. A higher score indicates more severe depressive symptoms. A total score ≥14 suggests clinically relevant depression (12). It is note that this classification reflects symptom severity and is not equivalent to a formal clinical diagnosis made by a mental health professional. In the present sample, the Cronbach’s α coefficient for the BDI scale was 0.927.

#### 2.2.4 Anxiety

We evaluated anxiety using the Self-Rating Anxiety Scale (SAS), which consists of 20 items rated on a 4-point scale (1 = none or occasionally, 4 = always). The raw score was multiplied by 1.25 to obtain the standard score; a standard score ≥50 indicates anxiety symptoms (13). As with the depression measure, this denotes a level of symptom severity based on self-report, not a clinical diagnosis. In the present sample, the Cronbach’s α coefficient for the SAS scale was 0.836.

#### 2.2.5 Sleep quality

We measured sleep quality using the Pittsburgh Sleep Quality Index (PSQI), which assesses sleep quality over the past month across seven components (sleep latency, duration, efficiency, disturbances, medication use, and daytime dysfunction). A global PSQI score >5 indicates poor sleep quality (14). In the present sample, the Cronbach’s α coefficient for the PSQI scale was 0.790.

#### 2.2.6 Insomnia

We measured insomnia using Insomnia Severity Index (ISI), a 7-item scale scored from 0 (no problem) to 4 (very serious problem). A total score ≥15 indicates severe clinical insomnia, with 10–14 (moderate) and 8–9 (mild) as subthreshold categories (15). In the present sample, the Cronbach’s α coefficient for the ISI scale was 0.794.

#### 2.2.7 Chronotype

We determined chronotype using the Morningness–Eveningness Questionnaire (MEQ), which classifies participants as “morning type,” “intermediate type,” or “evening type” according to their preferred sleep–wake pattern (16).

#### 2.2.8 Excessive daytime sleepiness

We evaluated daytime sleepiness using the Epworth Sleepiness Scale (ESS), which includes 8 items rated from 0–3. A total score >10 indicates excessive daytime sleepiness (17). In the present sample, the Cronbach’s α coefficient for the ESS scale was 0.841.

### 2.3 Data collection procedure

Data were collected from January to June 2025 using a structured questionnaire administered to clinical nurses. The questionnaires were distributed and collected by trained researchers during nurses’ working shifts. Before completion, participants received standardized instructions regarding the study objectives, confidentiality, and questionnaire completion procedures. Participation was voluntary, and all participants provided informed consent before completing the survey. The survey required approximately 25–30 minutes to complete. Completed questionnaires were reviewed by two independent researchers for completeness and data accuracy. Questionnaires with missing responses were excluded during data cleaning, resulting in a final analytical dataset without missing values.

### 2.4 Statistical analysis

Data were analyzed using IBM SPSS Statistics version 26.0 (IBM Corp., Armonk, NY, USA) and Jamovi version 2.3.2. The significance level for all tests was set at *p* < 0.05. Continuous variables were assessed for normality using the Shapiro–Wilk test together with visual inspection of Q–Q plots. Normally distributed variables are presented as mean ± standard deviation (SD) and were compared using independent-samples t tests, whereas non-normally distributed variables are presented as median and interquartile range (IQR) and were compared using the Mann–Whitney U test. Categorical variables are presented as frequencies and percentages and were compared using the χ² test or Fisher’s exact test, as appropriate.

Because most mindfulness subscales were not normally distributed, Spearman’s rank-order correlation analysis was used to examine the associations between mindfulness facets and psychological outcomes.

To identify factors independently associated with depression, multivariable logistic regression analyses was performed. Variables with *p* < 0.10 in the univariate analyses were considered candidate predictors to avoid prematurely excluding potentially relevant variables. In addition, clinically and theoretically important variables, including age, sex, marital status, education level, and professional title, were retained in the multivariable models regardless of their statistical significance in the univariate analyses to reduce potential residual confounding. Categorical variables were entered into the regression models using dummy coding. Adjusted odds ratios (ORs) and corresponding 95% confidence intervals (CIs) were reported.

To further evaluate the robustness of the primary findings, a sensitivity analysis was performed using multivariable linear regression with the continuous BDI total score as the dependent variable. The model included the same demographic, occupational, clinical, and mindfulness-related variables as the primary multivariable logistic regression model. Regression assumptions were assessed before model interpretation. Normality of residuals was evaluated by visual inspection of Q–Q plots, homoscedasticity was examined using residual-versus-fitted plots, independence of residuals was assessed using the Durbin–Watson statistic, and multicollinearity was assessed using variance inflation factors (VIFs). Heteroskedasticity-consistent (HC1) robust standard errors were applied to account for mild departures from residual normality and homoscedasticity. VIF values <5 was considered indicative of the absence of substantial multicollinearity. We further examined interrelatedness among sleep and anxiety predictors (PSQI, ISI, ESS, and SAS anxiety scores). Bivariate Spearman’s rank-order correlations between these variables ranged from *ρ* = 0.371 to 0.610, reflecting moderate conceptual overlap. In the full multivariable model, all VIF values ranged from 1.05 to 2.51, and no substantial violations of the regression assumptions were identified.

## 3 Results

### 3.1 Sample characteristics and univariate analysis

As detailed in **Table 1**, the study included 416 clinical nurses with a mean age of 31.3 years, the majority of whom were female (95.9%). The prevalence of depression was 56.0%, with poor sleep quality (54.6%) and clinical insomnia (52.4%) also highly prevalent. The elevated rates of depression and insomnia likely reflect the substantial occupational stress endemic to the clinical nursing population under study.

**Table 1:** Sociodemographic and occupational characteristics of participants (N = 416).

Variables	Category	n (%)/Mean ± SD
Sociodemographic factors		
Age (years)		31.3 ± 7.9
Sex	Female	399 (95.9)
Education level	Bachelor or higher	290 (69.7)
Marital status	Single	167 (40.1)
Occupational factors		
Professional title	Junior	298 (71.6)
Rotating night shifts	Yes	279 (67.1)
Work stress	High	261 (62.7)
Work interest	Low	309 (74.3)
Prevalence of key outcomes		
Depression	BDI ≥ 14	233 (56.0)
Anxiety	SAS ≥ 50	53 (12.7)
Insomnia	ISI ≥ 15	218 (52.4)
Poor sleep quality	PSQI > 5	227 (54.6)
Daytime sleepiness	ESS > 10	112 (26.9)

SD, standard deviation; SAS, Self-rating Anxiety Scale; ISI, Insomnia Severity Index; PSQI, Pittsburgh Sleep Quality Index; ESS, Epworth Sleepiness Scale.

Univariate comparisons between nurses with and without depression (**Table 2**) revealed significant differences (*p* < 0.05). Nurses with depression reported significantly higher work stress, lower work interest, more night-shift work, anxiety, insomnia, poor sleep quality, and daytime sleepiness. These variables, among others with *p* < 0.10, were considered candidates for the subsequent multivariate model.

**Table 2:** Comparison between nurses with and without depression.

Variables	Category/Description	Non-depressed (n = 183)	Depressed (n = 233)	Test statistic	*p*-value
Sociodemographic factors					
Age (years)	Mean ± SD	31.5 ± 7.8	31.6 ± 8.0	*t* = -0.695	0.487
Sex	Female	96.2%	95.3%	*χ²* = 0.06	1.000
Marital status	Single	37.7%	42.1%	*χ²* = 0.81	0.420
Education level	Bachelor or higher	69.4%	70.0%	*χ²* = 0.02	0.915
Occupational factors					
Professional title	Junior	75.4%	68.7%	*χ²* = 2.29	0.154
Night-shift work	Yes	77.9%	92.9%	*χ²* = 20.11	< 0.001^***^
Work interest	Low	62.8%	81.5%	*χ²* = 34.65	< 0.001^***^
Work stress	High	47.0%	75.1%	*χ²* = 17.59	< 0.001^***^
Health & lifestyle factors					
BMI ≥25 (kg/m²)	Yes	13.7%	12.4%	*χ²* = 0.134	0.770
Regular exercise	Yes	10.8%	8.6%	*χ²* = 0.65	0.503
Screen time ≥ 3 h/day	Yes	37.7%	51.9%	*χ²* = 8.36	0.004^*^
Caffeine intake	Yes	36.1%	44.6%	*χ²* = 3.12	0.088
Chronic diseases	Yes	35%	39.1%	*χ²* = 0.731	0.145
Traumatic experience	Yes	6.6%	17.2%	*χ²* = 10.55	0.001^**^
History of mental illness	Yes	2.7%	9.9%	*χ²* = 8.32	0.004^*^
Psychological & sleep outcomes					
Anxiety symptoms	SAS ≥ 50	4.9%	18.9%	*χ²* = 17.99	< 0.001^***^
Insomnia	ISI ≥ 15	30.1%	70.0%	*χ²* = 34.05	< 0.001^***^
Poor sleep quality	PSQI > 5	27.3%	76.0%	*χ²* = 97.83	< 0.001^***^
Daytime sleepiness	ESS > 10	13.1%	37.8%	*χ²* = 31.67	< 0.001^***^
Mindfulness (FFMQ), median (IQR)					
Observing		14.0 (12.8 – 15.0)		*U* = 19984	0.237
Describing		17.0 (16.0 – 18.0)		*U* = 19577	0.126
Acting with awareness		32.0 (31.0 – 34.0)		*U* = 1313	0.934
Non-judging		28.0 (27.0 – 30.0)		*U* = 19923	0.217
Non-reactivity		13.0 (12.0 – 14.0)		*U* = 18369	0.012^*^

SD, standard deviation; SAS, Self-rating Anxiety Scale; ISI, Insomnia Severity Index; PSQI, Pittsburgh Sleep Quality Index; ESS, Epworth Sleepiness Scale; FFMQ, Five Facet Mindfulness Questionnaire. Continuous variables were compared using independent-sample Mann–Whitney U tests, and categorical variables were compared using χ² tests. *p < 0.05 was considered statistically significant. *p < 0.05, **p < 0.01, ***p < 0.001.

### 3.2 Correlations between mindfulness and psychological outcomes

Spearman’s rank-order correlation analysis revealed distinct association patterns between mindfulness facets and psychological and sleep outcomes (**Table 3**). Among the five FFMQ facets, only non-reactivity was significantly and negatively correlated with depressive symptoms (*ρ* = -0.111, *p* = 0.024), indicating a potential protective effect. No other statistically significant bivariate correlations were observed between mindfulness subscales and the outcome variables (all *p* > 0.05).

**Table 3:** Correlations between mindfulness and psychological outcomes.

FFMQ subscales	Depression (*ρ*, *p*)	Anxiety (*ρ*, *p*)	Insomnia (*ρ*, *p*)	Sleep quality (*ρ*, *p*)	Daytime sleepiness (*ρ*, *p*)
Observing	-0.053, 0.280	-0.002, 0.969	-0.016, 0.745	-0.045, 0.357	-0.018, 0.715
Describing	-0.090, 0.066	0.029, 0.561	0.024, 0.629	-0.034, 0.490	-0.001, 0.984
Acting with awareness	-0.008, 0.868	-0.012, 0.809	0.095, 0.053	0.005, 0.920	0.047, 0.338
Non-judging	-0.051, 0.303	0.019, 0.694	0.031, 0.531	-0.020, 0.680	-0.069, 0.162
Non-reactivity	-0.111, 0.024^*^	0.042, 0.394	0.050, 0.307	-0.082, 0.095	0.069, 0.158

FFMQ, five facet mindfulness questionnaire. *ρ*, Spearman’s rank correlation coefficient. *p is statistically significant at the 0.05 level.

### 3.3 Multivariable logistic regression for depression

To identify factors independently associated with depression, a multivariable logistic regression analysis was performed. As shown in **Table 4**, nurses with a junior professional title, high work stress, prolonged screen time (≥3 hours/day), insomnia, and poor sleep quality had significantly higher odds of depressive symptoms. In contrast, higher mindfulness non-reactivity was independently associated with lower odds of depression (OR = 0.88, 95% CI = 0.79–0.98, *p* = 0.023). These associations remained significant after adjustment for demographic, occupational, lifestyle, and sleep-related covariates.

**Table 4:** Multivariate logistic regression analysis for factors associated with depression.

Variables	Adjusted OR	95% CI	*p*-value
Age (years)	1.02	0.97–1.07	0.364
Sex (female)	1.06	0.32–3.51	0.923
Marital status (single)	0.61	0.32–1.14	0.121
Education (bachelor or higher)	0.88	0.50–1.56	0.680
Junior professional title	2.25	1.09–4.65	0.029*
Night-shift work	0.857	0.48-1.50	0.592
Low work interest	1.76	0.99–3.12	0.056
High work stress	2.12	1.25–3.60	0.005*
Screen time ≥3 h/day	2.25	1.31–3.88	0.003*
Anxiety	2.01	0.79–5.07	0.141
Insomnia	2.15	1.22–3.75	0.007*
Daytime sleepiness	1.75	0.96–3.34	0.088
Poor sleep quality	4.19	2.47–7.12	<0.001*
Mindfulness: Non-reactivity	0.88	0.79–0.98	0.020*

The model was adjusted for all variables significant in univariate analyses (*p* < 0.10) and those of clinical relevance (e.g., professional title, education). Among the mindfulness facets, only ‘Non-reactivity’ was included as it was the sole dimension significantly associated with depression in univariate analysis. The model exhibits robust performance: Area Under the Receiver Operating Characteristic Curve (AUC) = 0.839 (95% CI: 0.79–0.88); McFadden’s pseudo R² = 0.289; Omnibus test: χ²=163, df=15, *p* < 0.001. OR, odds ratio; CI, confidence interval. *p is statistically significant at the 0.05 level.

### 3.4 Sensitivity analysis using multivariable linear regression

As a sensitivity analysis, multivariable linear regression was performed using the continuous BDI score as the dependent variable (**Table 5**). After adjustment for the same covariates included in the primary logistic regression model, higher mindfulness non-reactivity remained independently associated with lower depressive symptom severity (B = −0.509, SE = 0.150, β = −0.126, *p* < 0.001). In addition, poor sleep quality, anxiety, daytime sleepiness, prolonged screen time, low work interest, and high work stress were independently associated with higher BDI scores.

**Table 5:** Sensitivity analysis using multivariable linear regression with continuous BDI score as the outcome.

Predictor	B	SE	t	*p*	β
Constant	-8.134	3.667	-2.219	<0.027	
Age (years)	0.022	0.066	0.330	0.742	0.017
Sex (female)	2.083	1.792	1.163	0.246	0.209
Marital status (single)	-0.207	0.898	-0.230	0.818	-0.021
Education (bachelor or higher)	-0.108	0.815	-0.133	0.894	-0.011
Junior professional title	0.403	1.060	0.380	0.704	0.041
Night-shift work	-1.628	0.811	-2.004	0.056	-0.163
Low work interest	2.032	0.841	2.418	0.016*	0.204
High work stress	1.882	0.789	2.383	0.018*	0.189
Screen time ≥3 h/day	2.922	0.771	3.787	<0.001*	0.293
Anxiety	0.314	0.051	6.124	<0.001*	0.281
Insomnia	0.048	0.083	0.576	0.565	0.032
Daytime sleepiness	0.2445	0.0913	2.680	0.008*	0.129
Poor sleep quality	0.893	0.119	7.498	<0.001*	0.339
Non-reactivity	-0.509	0.150	-3.39	<0.001*	-0.126

The dependent variable was the continuous BDI total score. The model was adjusted for the same covariates included in the primary multivariable logistic regression model. The Durbin–Watson statistic was 2.13 (*p* = 0.212). Heteroskedasticity-consistent (HC1) robust standard errors were applied to account for mild departures from residual normality and homoscedasticity. All VIF values ranged from 1.05 to 2.51. B = unstandardized regression coefficient; SE = robust standard error; β = standardized regression coefficient.

## 4 Discussion

Our study highlights the substantial mental health burden among clinical nurses, with over half reporting clinically significant depressive symptoms (56.0%, BDI ≥14) and moderate-to-severe insomnia symptoms (52.4%, ISI ≥15). These prevalence figures, derived from standardized self-report screening scales, underscore the acute psychological distress within this frontline healthcare population and are consistent with some prior studies conducted in high-pressure nursing environments (18, 19). Rather than examining mindfulness as a whole, we investigated its multidimensional structure. Unlike the other four mindfulness facets, only non-reactivity remained independently associated with lower odds of depressive symptoms after multivariable adjustment, suggesting that different components of mindfulness may contribute unequally to psychological well-being among clinical nurses. This association remained significant when depressive symptoms were analyzed as a continuous outcome in the sensitivity analysis, supporting the robustness of the observed association across different analytical approaches. These findings shift the focus of mindfulness research from overall mindfulness to specific facets that may be more closely associated with depressive symptoms and suggest that non-reactivity may represent a promising target for future intervention research in this high-risk occupational group.

Non-reactivity refers to the ability to calmly observe internal experiences (thoughts, emotions, sensations) without being overwhelmed or reacting impulsively. Previous research in healthcare providers found that increases in non-reactivity were associated with reductions in perceived stress (20). In high-pressure nursing contexts, non-reactivity may act as an internal “emotional buffer” that helps separate stress-eliciting events from ensuing negative thoughts and emotions, thus preventing ruminative cycles or emotional exhaustion (21). For example, nurses with strong non-reactivity treat patients’ pain or relatives’ accusations as task-related scenarios rather than personal attacks, which enables quicker stress recovery and better emotional stability (22). This mechanism may partly explains why, in our regression model, non-reactivity remained an independent protective factor against depressive symptoms even after controlling for work stress and sleep disturbances. Although sleep quality, insomnia, daytime sleepiness, and anxiety are interrelated constructs, each reflects a distinct clinical dimension and may independently contribute to depressive symptoms. Therefore, these variables were retained simultaneously in the multivariable models to minimize residual confounding. Furthermore, multicollinearity diagnostics indicated no evidence of problematic collinearity, supporting the robustness of the estimated independent association between mindfulness non-reactivity and depressive symptoms.

This study has potential clinical and practical implications. The observed association between mindfulness non-reactivity and lower depressive symptoms suggests that non-reactivity may represent a promising target for future intervention development. Existing mindfulness-based approaches, such as the Three-Minute Breathing Space (23), have been reported to reduce burnout and compassion fatigue among nursing staff, and cognitive defusion exercises (e.g., Thoughts Are Just Thoughts) (24, 25) have demonstrated benefits in reducing the emotional impact and believability of negative self-referential thoughts. These interventions may provide useful directions for future research evaluating whether interventions specifically targeting non-reactivity can improve nurses’ mental health. Nevertheless, such individual-level approaches should be considered complementary to, rather than replacements for, organizational strategies such as optimizing shift scheduling and reducing workload (26).

This study has several limitations. First, the cross-sectional design precludes causal inferences between non-reactivity and depression; reverse causation is possible (e.g., depressive symptoms may impair non-reactivity). Second, all data were collected through self-reports, which may introduce common method bias. Third, the single-center sample may limit the generalizability of the findings. Future research should use longitudinal or interventional designs to clarify causality, include objective measures such as actigraphy for sleep, and test the results in a wider range of medical settings.

## 5 Conclusion

This study provides evidence that clinical nurses experience a substantial burden of depressive symptoms and related psychological distress. Among the multidimensional facets of mindfulness, non-reactivity was independently associated with lower odds of depression after adjustment for demographic, occupational, and clinical factors. This association remained significant when depressive symptoms were analyzed as a continuous outcome, supporting the robustness of the finding across different analytical approaches. These findings suggest that non-reactivity may represent a promising target for future longitudinal and intervention studies among clinical nurses.

## Data Availability

The raw data supporting the conclusions of this article will be made available by the authors, without undue reservation.
